# Challenges and understandings of creative practice in professional sport training

**DOI:** 10.1371/journal.pone.0279702

**Published:** 2023-02-22

**Authors:** Roberta Antonini Philippe, Michele Biasutti, Dylan van der Schyff, Andrea Schiavio

**Affiliations:** 1 Institute of Sport Sciences, University of Lausanne, Lausanne, Switzerland; 2 Department of Philosophy, Sociology, Education and Applied Psychology, University of Padova, Padova, Italy; 3 Melbourne Conservatorium of Music, University of Melbourne, Melbourne, Australia; 4 Centre for Systematic Musicology, University of Graz, Graz, Austria; Universiti Malaysia Terengganu, MALAYSIA

## Abstract

We conducted a qualitative study involving twelve expert sports coaches to explore and compare the range of creative practices they adopted during their professional activities. Their written responses to open-ended questions highlighted different interrelated dimensions of creative engagement in coaching sport, suggesting that efforts to instil creativity may initially focus on an individual athlete; they may often span a range of behaviours dedicated to efficiency; they may involve significant degrees of freedom and trust; and they cannot be captured by a single defining feature. We contextualise these findings in the light of recent literature in sports studies, performance science and creativity research, providing concrete examples based on the written statements provided by our participants. We conclude by offering insights for future research and coaching practice that may be relevant in broader domains.

## Introduction

Creativity, as a concept, has been a focus of discussion among many researchers who have analysed it in relation to their specific disciplinary fields. Notably, several scholars have criticised rigid conceptualisations and understandings of the concept of creativity in favour of a multidimensional vision [1–3]. Indeed, it has been argued that creativity is not a singular phenomenon or characteristic but takes many forms [4, 5]. It is also claimed that manifestations of creativity are driven by various *creative impulses* that undergo a form of “natural selection,” resulting in more stable forms of creativity [6]. Moreover, according to the multidimensional approach [7], creativity involves combinations of cognitive, conative, emotional, and environmental factors. Cognitive aspects such as personality traits and cognitive styles—i.e., an individual’s preferences for a given mode of sense-making—can influence the quantity and/or the nature of creative output. Conative factors are those aspects of mental processes or behaviour directed towards action or change, and they include impulse, desire, volition, and striving. The emotional sphere includes emotive states and moods, which often play an important role in characterizing creative potential and behaviour [7–10]. Environmental (i.e., social, cultural, educational, etc.) factors have also been associated with the ability to take risks and solve problems, thus being understood as fundamental drivers for human creativity and innovation [11–13].

Not surprisingly, creativity is at a premium in the context of sport and its complexity in relation to performance. Sport behaviours involve movements and motor variables that are unique in comparison to other creative acts. In a general sense, creativity is often considered as a set of (mental or physical) actions for producing innovative ideas, products, and behaviours involving novelty and efficacy. And indeed, reflecting such a complexity, motor creativity in sports has distinctive aspects; it involves the ability to bring to light something new and to create original physical movements that can lead to unique outcomes [14, 15]. The relationships between different environmental, personal, and performance conditions show that the problem at hand always has more than one effective solution possible and that the effectiveness of those solutions is not pre-determined [16, 17]. How can we capture this open dimension of creative action? On the one hand, the cognitivist tradition has been at the forefront of creativity research, theorizing creativity in terms of information processing that plays out in the brains of creative agents [18]. On the other hand, recent understandings of mind and subjectivity based on embodied cognitive science [19–21] offer novel conceptual tools to help us rethink the role of bodily activity for mental life, favouring in turn the development of innovative understandings of creativity [22, 23] and skilled action [24–26] that include corporeal and environmental aspects as key components.

The liaison between these orientations, as we will see, has implications for the conceptualisation and study of creativity in sport. Here, as the concept of creativity develops certain variables are of more interest to researchers, such as the development of expertise and individual talent [27], tactical creativity [28–33], as well as attention and creativity [34–37].

Other variables associated with sport include interventions or, more precisely, training programmes that help creativity to flourish [38–40]. According to Jowett’s (2007) theoretical framework, coach–athlete relationships are defined as social situations created by the interplay of the coaches’ and the athletes’ interpersonal feelings, thoughts, and behaviours [41]. A recent systematic narrative review [42] found few studies that had examined coaches’ perceptions of creativity and its development in their athletes. The studies that were found focussed on aspects such as beliefs about teaching [43] and the nature of creative thinking [44]. Using questionnaires, Oh and colleagues (2010) found that coaches associated their own creativity with variables such as “adaptability, improvisation, and mediating” (p. 65) [43]. That study showed that their fundamental skills and self-determination were important tools for developing creativity. However, coaches seemed to be unprepared to teach creativity and trusted their own experience more than using ready-made recipes. Another study used a questionnaire with closed-ended questions [44] and showed that there were statistically significant differences in most creative concepts related to coaches’ perceptions of creativity. Apparently, coaches mostly associated creativity in soccer with a kind of “magical thinking”—the intelligent ability to be rational, solve problems, and make decisions.

Theoretical models (see [41] for a review) have shed light on the components of these interpersonal relationships that are important to consider when working with athletes. In addition, training programmes have also evolved not only in the areas of technical, technical, and physical improvement but also by taking into account the more interpersonal variables of the relationship. However, much remains to be understood about this complex phenomenon in the domain of coaching. Indeed, environmental dynamics and non-linear interactions between system components provide an important framework within which to understand the various connections between a person and their environment as they also search for adaptability and actual creativity [16, 45].

### Aims and research questions

The present study was part of a larger research project devoted to investigating the role of creativity in various teaching and learning contexts. This research aimed to build a detailed picture of the challenges and understandings of the creative engagement that professional sports coaches experience in their everyday activities. To address this aim, we used a qualitative technique based on questionnaires with open-ended questions sent to our participants via e-mail. This approach provided participants with more time to reflect and to express their thoughts than other qualitative methods, such as interviews. This can be beneficial when respondents are asked to describe situations in detail and offer concrete examples. A similar questionnaire-based protocol was successfully implemented in previous research exploring closely related topics [46, 47]. The present study’s two key research questions were:

(1) How do professional sports coaches conceptualise creative practice?
(2) What strategies do professional sports coaches use to enhance creativity in their athletes?

### Methods

#### Participants

Participants were recruited through an announcement on social media and via personal contacts. The final sample included twelve volunteers (seven men; five women), a size comparable with similar qualitative studies [48]. Our respondents were aged from 30–48 years old (mean age = 39.8; SD = 5.10), and all had at least two years of professional experience as the head coach of different sports teams, including water polo (2), sailing (2), figure skating (2), diving (1), rugby (2), basketball (2) and soccer (1). These details are summarised in Table 1.

**Table 1:** Overview of the participants.

	Age	Gender	Sport	Years of coaching
**C1**	48	M	Water polo	7
**C2**	38	M	Sailing	16
**C3**	37	W	Figure skating	17
**C4**	37	M	Diving	7
**C5**	36	M	Rugby	3
**C6**	41	M	Basketball	20
**C7**	40	M	Soccer	2
**C8**	45	M	Rugby	10
**C9**	48	W	Sailing	7
**C10**	33	W	Figure skating	2
**C11**	36	W	Water polo	10
**C12**	30	W	Basketball	8

## Materials and data collection

The research team designed a two-part questionnaire of open-ended questions (available on request from the corresponding author). The first part involved general questions related to demographics, personal background, and professional interests; the second part featured 11 items addressing our two key research questions and focussing on several interrelated topics. Examples of questions include: “What does ‘creativity’ mean to you?”; “How do you cultivate creativity in the athletes you train?” and “How do your athletes react when engaging in creative practice?” The questionnaire was sent to participants via e-mail, in English, French or Italian, according to their preference. Participants were instructed to respond to each item discursively and to return the completed questionnaire within two weeks. The coaches were based in Italy, France, and Switzerland and gave their written informed consent before taking part in the study.

### Data analysis

This study adopts hermeneutic phenomenology as the research approach. Hermeneutic phenomenology aims to analyse experience by describing a phenomenon in terms of how it emerges from the fringe of consciousness and how it affects individuals on a personal and social level. Each coach was assigned a code (i.e., C1–C12) to ensure anonymity. A thematic inductive approach was employed to analyze the data, which involved different steps [49]. The transcripts were first read through to familiarize the researchers with their content. Analysis involved identifying and dividing the transcripts into meaning units: parts of text representing a single idea in relation to the research question. These meaning units were labelled and then reviewed across all of the transcripts to check for consistency across the dataset. The meaning units were grouped into categories and themes with other similar meaning units. This procedural step helped to define a preliminary thematic categorisation of the responses, which was then completed by a more in-depth thematic analysis that revealed four thematic units: ‘*The creative individual*’, ‘*Towards efficiency*’, ‘*Freedom and trust*’ and ‘*The many sides of creativity*’.

Steps were taken to establish and ensure the reliability of the analysis process. We employed several techniques to ensure the trustworthiness and credibility of the data. First, all four researchers were familiar with qualitative analysis. Second, we took a collaborative approach in the process of data analysis to reduce interpretive bias. Two members of the research team (first and last authors) independently identified and coded meaning units in each of the interview transcript and, subsequently, engaged in several reflective discussions to ensure agreement in the process of interpretation and the coding of themes. This ensured coding reliability (inter-rater reliability) and minimized interpretive bias [33]. The process of reflection and verification between the researchers continued until all themes were agreed and verified, after which time, final themes and categories were established. Both researchers were expert in sport domain.

The study was carried out in accordance with the Declaration of Helsinki and the Code of Ethics and Conduct of the British Psychological Society. Written and informed consent was obtained from all participants attesting that the data could be analyzed and discussed for publishing. Ethical approval for the recruitment of questionnaire respondents was granted by the Research Ethics Committee of the University of Graz (GZ. 39/17/63 ex 2020/21).

## Results

This section reports and contextualises our participants’ written descriptions of how different dimensions of creativity permeated their discipline. Statements and examples associated with different training and performance experiences highlighted how creative efforts might initially focus on the individual athlete; those efforts often span a range of behaviours dedicated to efficiency; they may involve an important degree of freedom and trust; and they cannot be captured by any one defining feature.

### The creative individual

When coaches were asked about creativity in practices and training, many of their statements appeared to focus on the individual athlete and on the rich variety of techniques used to enhance their creative efforts:

“[Creativity] means allowing a given person to express herself, interpreting, adding, appropriating a given item, object, assignment or exercise, whatever the context. Creativity is personal, intimate and subjective.” (C1)

This quotation arguably highlighted two key points: firstly, the relationship between coach and athlete, and secondly, the athlete’s personal response to the use of a context-specific item or demand. Regarding the relationship, it is interesting to note how the coach used the verb “to allow” to note the constraints that creative activity involves: in sports, as in other domains, creativity is not simply about acting freely. Instead, one must consider the strategies, suggestions and plans put forward by the coach to achieve a specific creative goal within the constraints of the sport being performed (e.g., rules, established, techniques, strategies, and so on). The second focal point individuated above, that is, promoting the athlete’s personal reaction, might involve finding the right balance between an ‘intimate’ dimension (e.g., an innovative personal decision taken during a group training exercise) and a contextually meaningful outcome (e.g., improving group efficiency during that exercise). For this interplay to be functional, it is vital that the athlete becomes aware of their role in the process, as mentioned by other participants.

“Creativity is about knowing oneself and becoming aware of oneself in order to create, produce and renew oneself.” (C5)

“For me, creativity means creating something potentially new, or at least out of the ordinary, for ourselves. It is closely related to the notions of creation and innovation.” (C8)

Note the focus on *individuality* in both quotes. Perhaps both the athlete and the coach should work consistently on individual talents, leading to a range of diverse, fine-tuned outcomes that might play out collectively. This possible dialectical relationship between the individual and contextual dimensions was captured by the following statement.

“I promote autonomy, social proximity and the notion of perceived competence. I work on the individual levers of motivation, singular to everyone, that allow them to be creative in [given] domains. This can be self-help or solidarity as well as the team’s competitive spirit.” (C3)

Here the coach described what might be defined as a continuum between individual and collective creative effort: not only can individual work enhance the athlete’s motivation and expertise; it can also have an important impact on the rest of the team. Ultimately, the focus on a single athlete, reported to be at the heart of training efforts, may be seen as a fundamental aspect of what being together in a team really means, as changes in the former will inevitably influence the latter. But although a coach can use multiple strategies to stimulate individual and collective creative outcomes along this continuum, there remains an important difference between solo and joint creativity:

“Groups become creative with practice, but a talented player is naturally more creative. There are cases where one needs to be creative on one’s own and act quickly, and this is difficult to coach.” (C6)

Perhaps this statement provides a good account of why a major focus can initially be placed on individual athletes and the changes they can bring to a team: sometimes skilled individuals may require less training than others to act creatively, providing a positive influence on the rest of the team. To further illustrate this point, another participant provided this concrete example.

“Water polo is about creativity: there are the rules of the game, there are techniques, drills, etc. However, that creativity is within each athlete, from the way [they] pass the ball to the way they achieve their position in the water. They have to find their own style and be comfortable when shooting. And one has to create one’s own position in the water.” (C1)

While this quotation points again to the role of individual players, it also suggests that their creative efforts always remain situated within the social, contextual dynamics of action. If we briefly consider how the bodily positioning each player needs to achieve in the water is dynamically influenced by both teammates and opponents, then individual and collective dynamics are inherently intermixed here. This is because creative behaviours, such as passing the ball or finding and maintaining a critical location in the pool, are determined by the constant flow of (inter)action the game entails. As we see below, this often involves a good deal of goal-oriented effort.

### Towards efficiency

This thematic category was characterised by descriptions pointing to an outcome-oriented understanding of creativity in both training and performance. Consider, for example, the following definitions offered by three different participants who identified creativity with “a way of searching for a result, via a task, by breaking the routine and re-inventing it” (C7), with an “alternative way of getting to the solution of the problem” (C4) and “a way of improving: bringing solutions while having fun” (C11). Focussing on the goal(s) of creative effort is an essential component of what creativity entails, along with its innovative features. This dual emphasis on functionality and innovation was well captured by another participant.

“A player needs to develop something unexpected and positive while playing. It is important that the team benefits from this; otherwise, there is no real value in it, and you can actually make a fool of yourself.” (C6)

This quotation explicitly referred to “value” as a fundamental category that involves both the individual and the team: the athlete’s creative effort, in this view, should be thoroughly dedicated to the team’s overall success. As a basketball coach, C6 described this sport’s intrinsic collaborative spirit well, illustrating the negative consequences that may emerge when no tangible benefits are provided to the team. An example of this might be a player performing a spectacular slam dunk during a fast break towards the end of a game where their team is trailing by 50 points: the slam dunk’s innovative or surprising characteristics serve no precise purpose given the score—they, therefore, provide no evident benefits to the team. In fact, this action may even be detrimental to the athlete, as their teammates may think that they were just attention-seeking rather than remaining focussed on the game. It should be noted that similar situations may not only occur during games; training sessions are also important occasions during which individual talents should not only be showcased but also put to the service of the team.

“In sport, creativity should not look like ‘stupidity’. Exercises or training processes should initially be solution-oriented. Creative exercises or solutions should not [replace] health or safety.” (C4)

This last statement emphasised the goal-oriented component of creative practice in sports but also highlighted its limits: one can only explore new creative solutions if the process is safe both for the athlete and their teammates. In tackling creativity in these terms, our respondents also placed a major emphasis on the balance between novelty and appropriateness, suggesting that one must be(come) aware of the various functional constraints that might impede or further enhance their creative drive. It should also be noted that while it is often the athlete who takes the initiative to explore novel creative possibilities, in many cases, the coach shares this responsibility too. By helping athletes navigate the range of creative landscapes, coaches play a fundamental role in both problem-finding and problem-solving processes.

“They are a bit shy at first because they are not used to being an actor in these processes (finding feedback…), especially the younger ones. The younger ones lack experience of that, so I don’t insist on it too much, and I make it easier for them by guiding them through the process by simplifying the reflection (example: do you think it was better or not?) and by transmitting the notions and keywords specific to the discipline.” (C9)

And as an important resource for creative flourishing (and flourishing creatively), “You have a chance to continue growing as a coach as well.” (C4) This last point resonates well with quotations from other participants:

“The happiness that comes from seeing people fulfilling themselves through the creative process [is the best part of training]. It fuels me, personally, to be able to create the right environment for everyone to fulfil themselves through the creative process.” (C3)

Note how this statement also pointed to the ongoing dialectics between creative production and the surrounding social environment. Here, the process of thriving creatively not only involves teammates but extends to the coach and their role in supporting each athlete. As such, the dimension of creative efficiency can be seen from a distributed perspective, whereby the whole team—including the coach—participates in nurturing creativity.

### Freedom and trust

We saw above how some athletes are thought to display a more natural inclination to acting creatively than others. Ideally, this tendency should not be a distraction, but play an important role in guiding the whole team towards different creative outcomes, stimulating others to act creatively too. This is particularly important in team sports, as one coach emphasised:

“I make [the players I coach] interpret my instructions very flexibly. When there is a play that needs to be executed at a certain time, or when I make a suggestion during a game, I do not get angry if they find a new solution or do something else. After all, they are there playing, and they can feel the game very differently from me, so they get certain details that I miss. Training this is hard, but trusting your players is the key to being a good coach.” (C6)

Trusting in the whole team, despite their differences and skill levels, is undoubtedly a good step towards establishing an efficient environment. As such a space for creative development is described, coaches can reflect on what role they actively play in training creativity:

“Honestly, I don’t think I have ever explicitly or systematically cultivated creativity in the athletes I have trained. It was more of a spontaneous process, mainly driven by curiosity—like trying something that nobody had done before (to the best of my knowledge). Sometimes it was some new challenge, keeping the competitive spark alive; other times it was about breaking out of patterns, asking them for something *outside the box*.” (C2)

“I never asked myself that question. But the fact that I let them have a lot of freedom in the training sessions was a strength for the development of creativity. In basketball you have to be creative, you have to be able to invent and that’s what I try to do by offering them fairly open training sessions.” (C12)

This statement suggested that creativity may span a wide range of domains, including how coaches engage with their athletes. Another participant supported this point well:

“I can give the framework, that is, the theme of the session (e.g., proprioception) as well as the number of sets, reps, work and break times, and I let the athlete organise himself to go after those goals. This means that he or she will go and get the material necessary to carry out the imposed thematic series. […]. Within this framework, the athlete is free to create, innovate, produce new exercises that are still adapted to the context of the session. This is made all the easier by integrating the skaters’ artistic aspects.” (C3)

As another participant suggested, the need for social engagement and participation may also lead to positive benefits in everyday life.

“The best thing is to [be] open-minded […] for a large part of the players, depending on their feelings and individual or collective perspectives. They need to understand that they are not alone and that they can be creative [to achieve] a positive result and continue to improve […]. Creativity inspires plenty of values for life.” (C5)

Coaches consistently referred to the social values of efforts to be creative, suggesting that a good training environment need not be governed by a rigid master–apprentice-type relationship. Instead, players and coaches can reciprocally affect each other’s potential for creativity by trusting in each other and leaving mutual space for action. This in no way implies that coaches lose their roles as guides, as one coach commented.

“As far as I am concerned, I do not see creativity as being the opposite to discipline or enforcing the coach’s guidelines or tactics.” (C1)

“In order to develop creativity, it is necessary to have a good relationship and commitment with the skaters. The motivational climate is important and it is in this type of climate that the skaters can express themselves more.” (C10)

### The many sides of creativity

We have seen how our respondents illustrated various aspects of their efforts at creativity in coaching sport via references to individuality, efficiency, trust, and freedom. Besides these dimensions, however, surprise can also be understood as another significant property of creativity. As one participant suggested, “Creativity [is] always accompanied by surprise.” (C6) The sense of surprise that often stems from a creative action—e.g., when completing a given task despite having deviated unexpectedly from the coach’s precise instructions—can help athletes and coaches reflect on their respective roles.

“I think creativity helps to keep people from taking themselves too seriously, which is important when you are under pressure. It helps to open the mind, which is important in critical decisions. Finally, disrupting patterns helps to detect weaknesses.” (C2)

Not only does this statement echo a previous quotation from C5—concerning the possible values that creativity might inspire in our lives—it also suggests another possible function: detecting weaknesses. This detection of weaknesses is seen positively by coaches, as it allows the athlete to step out of the comfort zone and look for new solutions. This arguably reflects the complexity of the very notion of creativity itself, which encompasses a rich variety of contexts, situations, and states of mind. Illustrating this, one coach explicitly described how reducing the notion of creativity to just one dimension would be impossible.

“I cannot define creativity as one thing and one thing only […]. When I think about this sport [basketball], I can distinguish at least four different ways of being creative: offensively alone, offensively with the team, defensively alone, and defensively with the team. Each has very different qualities.” (C6)

To support this statement, the same coach provided a few personal examples of what creativity might entail for him.

“Being a creative coach could mean many things: how to support your players, how to invent a play on the spot, understanding when to call a timeout, etc.” (C6)

According to this coach, any definition of creativity would inevitably be too reductive to capture the phenomenon’s full complexity. However, other coaches provided complementary insights highlighting a range of basic principles, including:

“The ability to appropriate, add, personalise a drill, a given game-situation; the ability to think differently or wanting to try new things.” (C1)

“It`s just to give them freedom but with guidelines, for example, training situations where they can express themselves and use their creativity.” (C7)

But another coach unpacked the importance of exploration, with insights bringing us back to focus on efficiency, as addressed earlier.

“I have the impression that some creative ideas come from a kind of need to keep exploring. […] So, I’d not say that [creativity] comes from […] inspiration; it’s more about having an underlying problem to solve and finding the solution when it’s possible to stay on the edge of the consciousness.” (C2)

## Discussion and conclusion

The present study aimed to explore the challenges and understandings of engaging in creativity as perceived by professional sports coaches in their experiences of everyday coaching activities. Several insights appeared in our findings regarding coaches’ conceptions of creativity and their strategies for enhancing creativity in their athletes.

With regards to its conceptualisation, “creativity” involves a sphere of multiple interactions, resulting in multiple meanings. Some of the statements reported in this study aligned well with research that emphasises how creativity is based on innovation and functionality [50]. Instances of creative thinking and behaviours were associated with both personal and contextual dynamics, as well as with the ability to develop (novel and valuable) training and competitive outcomes driven by a desire to explore [51]. Participants expressed a view that creativity is not just a goal to be reached but is also a process or method of reaching one’s full potential [52]. Here, creativity is interpreted as a crucial component of human development, bringing together several different aspects ranging from the importance of problem-solving to the role of bodily movement in creative efforts. In addition, participants reported how a particular state of mind could be beneficial in many contexts or situations prone to creativity. They conceptualised creativity as being multidimensional and mediated by interpersonal relationships [53], highlighting the deep connection between the pursuit of human potential and sociality [54, 55].

Regarding the strategies implemented to develop creativity, our findings were congruent with other coach–athlete relationship studies [41, 56], suggesting that the quality of interpersonal relationships is a crucial condition for creative flourishing and for implementing a creative coaching style. In most instances, athlete and coach operate together within a framework with its own unique characteristics and performance imperatives; trust and freedom appear to be necessary elements for creating a beneficial training environment within that framework, and for the development of creative skills across the whole team. Athletes are driven towards setting up mechanisms to help them adapt to their coach’s requirements to deploy the necessary creativity in action.

The ability to adapt remains a fundamental construct within teams, necessary to ensure their equilibrium [57, 58]. This capacity appears to be a condition for coaching creatively—an ability to not rely exclusively on rigid norms or patterns. Instead, adaptation develops in a similar way to how players perform during a game: exploration and novelty play a key role here too, as does the ability to take a step back and give athletes enough freedom to navigate the possible creative outcomes that thinking and acting outside of the box might entail.

Various studies [56, 59, 60] have highlighted the importance of interpersonal relationships in the quest for optimal performance, including a shared commitment to constant improvement. These studies underlined the coach’s decisive influence on the outcome of the athlete’s personal efforts through their influence on the athlete’s passion for practise or training. The present study’s novelty lies in its descriptions of how developing engaged, passionate practice sessions also seems to be linked to the creativity exhibited by the coach.

Before concluding, we note that our study had some methodological limitations: participant sampling might have been biased by the stressful or difficult periods during which they filled in the questionnaire (e.g., injuries, personal circumstances, the global pandemic), or by the career aspirations that could have influenced their answers. Although the number investigated in this study was only 12 coaches, we can assume that grounded in recent arguments [61], our results can be generalized related on the rigorous methodology in data collection and their analysis.

In conclusion, the findings stimulated several reflections, which could have educational implications and that call for further research. We suggest that future work explores the different facets of creativity in particular environmental contexts to better understand how to harness athletes’ human potential through learning and development programmes. Further, individual and team sport activities could be the focus of additional studies for highlighting how creativity unfolds. Finally, the theme of interpersonal relationships in sport seems to be an area for further research. This theme has been extensively investigated in the field of leadership and cohesion, but few studies have focused on creativity. In particular, it would be interesting to combine quantitative methods with more qualitative methods, for example focus groups, in order to better understand the development of creativity through interpersonal relationships.

## Supporting information

S1 File (DOCX)
