# Supplementary material for: Challenges and understandings of creative practice in professional sport training
Source: PLoS One. 2023 Feb 22;18(2):e0279702. doi: 10.1371/journal.pone.0279702 (PMC9946216; doi:10.1371/journal.pone.0279702)
Supplement: S1 File — (DOCX) [file pone.0279702.s001.docx]

**Study**: “Creative skill development”

**Contact**:

Dr. Andrea Schiavio: [andrea.schiavio@uni-graz.at](mailto:andrea.schiavio@uni-graz.at)

In this study we investigate how expert trainers and teachers think about, and engage with creative practice. We thus ask you to complete an open-ended questionnaire. Please note, all responses will be kept confidential. This means that your responses will only be shared with research team members and we will ensure that any information we include in our report does not identify you as the respondent. Remember, you don’t have to write about anything you don’t want to.

Please write YOUR INITIALS in each box

1. I confirm that I have read and understood the information explaining the research project and I have had the opportunity to ask questions about the project.
2. I understand that my participation is voluntary and that I am free to withdraw at any time without giving any reason and without there being any negative consequences. In addition, should I not wish to answer any particular question, I am free to decline, notifying the experimenter afterwards.
3. I understand that my responses will be kept strictly confidential. I give permission for members of the research team to have access to my anonymised responses. I understand that my name will not be linked with the research materials, and I will not be identified or identifiable in the report or reports that result from the research.

4. I agree for the data collected from me to be used in future research.

1. I agree to take part in the above research project.

________________________ ________________ ____________________

Name of Participant Date Signature


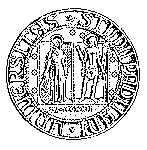

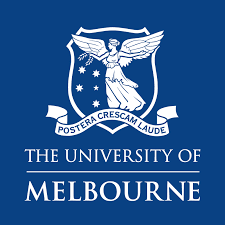

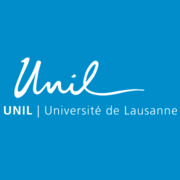

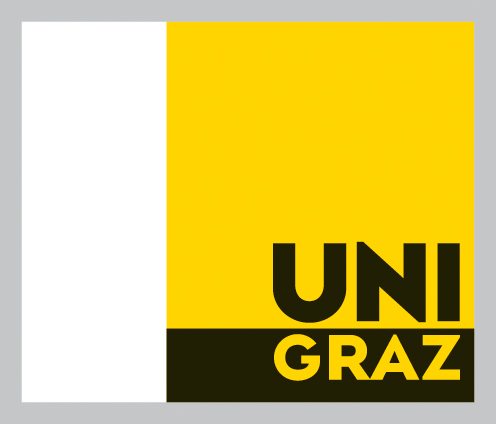


University of Graz, Austria

University of Padua, Italy

University of Lausanne, Switzerland

University of Melbourne, Australia

**Questionnaire for sport coaches**

The following interview/questionnaire aims to gather relevant information on how teachers and coaches think about, and engage with creative practice. This was developed by researchers from the University of Graz (AT), the University of Padua (IT), the University of Lausanne (CH), and the University of Melbourne, (AU).

**Part A: General Information**

# Age: ____ Gender: M ⁭ F ⁭ Other ⁭ _________________________________________________

Main sport played: _______________________________________________________________________

Your role (if any): ________________________________________________________________________

Favourite sport to watch: __________________________________________________________________

# What do you coach and where?

_______________________________________________________________________________________

For how many years have you been coaching? _________________________________________________

How many hours do you train athletes/teams per week approximately? ______________________________

How many athletes have you coached approximately? ___________________________________________

**Part B: Questionnaire**

# Please provide detailed answers to the questions below, without leaving out anything - including aspects that might be considered trivial. Where possible, please feel free to add specific examples from your own experience. Please note, this questionnaire is completely anonymous. It does not evaluate aspects related to intelligence or expertise. It only aims to gather information on how you think about and experience your creative work. There is no word limit - you are free to write as much as you want.

1. What does “creativity” mean to you?
2. How do you cultivate creativity in the athletes you train?
3. On what principles do you consider something being creative or not?
4. Please provide an example of a creative training you use for your athletes.
5. How do your athletes react when engaging in creative practice?
6. What are the main differences between training creatively individuals or groups?
7. What inspires you to be creative?
8. What are the main differences and continuities between creativity in music and creativity in other domains (e.g., music)?
9. What is the best aspect of creative coaching and practising? Why?
10. What is the worst aspect of creative coaching and practising? Why?
11. Would you like to add anything else?
